# Editorial: Multitasking: Executive Functioning in Dual-Task and Task Switching Situations

**DOI:** 10.3389/fpsyg.2018.00108

**Published:** 2018-02-15

**Authors:** Tilo Strobach, Mike Wendt, Markus Janczyk

**Affiliations:** ^1^Department of Psychology, Medical School Hamburg, Hamburg, Germany; ^2^Department of Psychology, Eberhard Karls University Tübingen, Tübingen, Germany

**Keywords:** dual tasking, task switching, executive functions, task coordination, task preparation

Persons are often engaged in activities that combine multiple tasks (so called *multitasking*), even though this combination is typically accompanied by performance costs in the individual tasks in comparison to their performance as single tasks. Such performance costs suggest that performing multiple tasks brings the cognitive processing system to its limits. However, the observed limitations can inform theories of how cognitive processing is generally organized. In other words, investigations on the limitations of multitasking performance can reveal fundamental aspects of the cognitive processing architecture and mechanisms of human information processing.

These aspects have been investigated with a variety of experimental paradigms typically comprising two different component tasks that vary in the degree of temporal overlap. While it is difficult to define with precision what constitutes a “task” (Rogers and Monsell, 1995; Monsell, 2003; Kiesel et al., 2010), one can define “task” broadly, so that (i) simple stimulus-response (S-R) translations [e.g., press a response key when hearing a low tone in so-called choice reaction time (RT) tasks], (ii) continuous tasks like motor tracking, (iii) complex movements (e.g., type writing), or (iv) tasks without necessarily yielding overt behavior (e.g., counting) can constitute a task if a person aims to achieve a discriminable goal state. Irrespective of the specific type of task, multitasking research includes research on dual-task performance and task switching performance (Pashler, 2000). While dual-task performance requires concurrent and simultaneous task processing and motor responses, task switching focuses on multitasking with sequentially processed component tasks.

## Dual-task paradigms, theories, and executive functioning

Generally speaking, there are two paradigms to investigate dual-tasking. In the simplest version, dual-task performance is compared with single-task performance, with only one stimulus/task being presented in the latter condition. Notably, in this paradigm, there is either a single stimulus or two simultaneous stimuli at the same time, and task load is manipulated in a one-vs.-two manner. In other words, participants either perform one task or two tasks per block. Dual-task performance costs are reflected in worse performance in dual- compared with the single-task conditions (e.g., Fagot and Pashler, 1992; Huestegge and Koch, 2009)^1^.

A second dual-task paradigm employs (most often) two choice RT tasks and varies the amount of temporal overlap of the two tasks. This *overlapping task paradigm* is nowadays often referred to as the Psychological Refractory Period (PRP) paradigm (Welford, 1952; Pashler and Johnston, 1989; Pashler, 1994). More specifically, stimuli of the two tasks are presented in a predictable order separated by a variable stimulus onset asynchrony (SOA). With short SOA (e.g., 50 ms), task overlap is high while with a long SOA (e.g., 1,000 ms) task overlap is low. Typically, RTs of Task 2 increase, the shorter the SOA between both tasks are (i.e., the PRP effect; see Janczyk et al., 2014, for exceptions to the PRP effect), while the SOA has no or only a small influence on RTs of Task 1 (see Strobach et al., 2015, for more information on Task 1 data and results).

In particular to explain the PRP effect, the prominent *central bottleneck theory* (Welford, 1952) holds that the selection of a response cannot be made for two tasks in parallel, while the initial perception stage (during which stimulus information is processed) and the final motor response stage (during which the motor response is executed) can run in parallel. Thus, response selection is conceived as a structural and unavoidable central processing bottleneck, leading to a long interruption of Task 2 processing at short vs. long SOAs and, hence, the PRP effect (Pashler, 1994). According to other bottleneck theories, a bottleneck exists in the motor response stage, preventing two responses from being initiated simultaneously or in close succession, as an alternative to the response selection bottleneck or in addition to it (e.g., De Jong, 1993; Sigman and Dehaene, 2006; Bratzke et al., 2009).

*Resource theories*, in contrast, assume that the critical capacity-limited stages can run in parallel, but as they share a common and limited attentional resource, this processing is less efficient compared with a single-task condition (e.g., Navon and Miller, 2002; Tombu and Jolicœur, 2003; Wickens, 2008). As was shown by Navon and Miller (2002) and Tombu and Jolicœur (2003), such capacity-sharing models can in fact explain many of the phenomena usually taken as evidence for bottleneck models. Further, if all capacity is first devoted to Task 1 and then to Task 2, the models mimic essentially a bottleneck model, which can thus be seen as a special case of capacity-sharing models.

Several studies also used variations of the PRP paradigm for analyses of executive control functions (Jiang et al., 2004; Strobach et al., 2012, 2014), for example, PRP experiments in which the order of the two tasks was not predictable (Sigman and Dehaene, 2006; Kamienkowski et al., 2011; Riuz Fernández et al., 2011; Hendrich et al., 2012; Töllner et al., 2012). The executive functions thought to be involved in performing such tasks are conceived as general-purpose control mechanisms that regulate the dynamics of human cognition and action (Miyake et al., 2000; Miyake and Friedman, 2012). In the context of dual-tasks, such control mechanisms coordinate the processing of two simultaneous task streams and the access to capacity-limited processing stages (e.g., De Jong, 1995; Luria and Meiran, 2003; Sigman and Dehaene, 2006; Szameitat et al., 2006). Exemplary empirical evidence for the flexible access to capacity-limited stages comes from the observation of a general increase of RTs for Task 1 in PRP dual-task RTs compared to single-task RTs, which points to the implication of time-consuming coordination processes at the beginning of dual-task trials (e.g., Jiang et al., 2004). From a perspective of executive processes, dual-task performance data may thus point to a set of well-identifiable task coordination processes. Recent studies investigated, for example, the impact of practice (e.g., Strochbach and Schubert, 2017), age (e.g., Maquestiaux, 2016), compatibility of stimulus and response information (e.g., Hazeltine et al., 2006), or recently experienced conflict (e.g., Janczyk, 2016) on dual-task performance and executive functioning in dual-tasks. In the following section, we provide a brief overview on papers of the present research topic aiming to contribute to the further specification of executive functions implicated in dual-tasking.

## Dual-task studies in the present research topic

Hommel et al. investigated the impact of binaural beats on cognitive flexibility to control two simultaneous tasks with overlapping task information in the PRP paradigm. Their findings showed that binaural beats can modulate the flexibility of executive control functions in dual-tasks. Thus, this method has the potential to bias the executive control style in dual-tasks. Schubert et al. investigated the contribution of dual-task coordination skills to a reduction of dual-task costs as a result of practice. The authors showed that these skills are fully independent from practice situations and are transferable to new dual-tasks. Pieczykolan and Huestegge investigated whether flexible control of dual responses varies depending on task complexity, manipulated as the number of task-relevant response combinations and the to-be-retrieved S-R translation rules. Their findings showed that the increase of both, response combination and the S-R translation rules as well as their preparation yielded an increase of dual-task costs. In sum, the findings stress the importance of memory retrieval processes in dual-response control.

From an aging perspective, it is known that older adults are particularly impaired in dual-tasks compared with single-tasks and young adults (e.g., Verhaeghen et al., 2003; Verhaeghen, 2011). Therefore, it is relevant to investigate why older adults are impaired in dual-task situations and whether they are particularly impaired in these situations' executive control functions. In a real-world task setting, Stelzel et al. investigated the characteristics of this impairment with a specific focus on the compatibility of input and output modality pairings in the two tasks. They demonstrated that dual-task postural control is impaired in older adults in contrast to young adults particularly with incompatible input-output modality pairings. A real-world task was also applied by Steinborn and Huestegge who combined mental arithmetic and phone conversation in a continuous dual-task paradigm. In the context of their attentional-failure account, they showed that mental arithmetic affected different aspects of phone conversation: information processing in participants' conversation was particularly slowed down for controlled processing components in comparison to automatic components. de Tomasso et al. analyzed electroencephalic and electromyographic responses in a passive auditory oddball paradigm for both patients with Huntington's Disease and healthy controls. A similar increase in the amplitude of the P3 component was observed for both groups when auditory stimulation was presented in dual-task situations with walking. Finally, Xing and Sun applied a dual-task situation to characterize rule-based category learning. These authors showed that the effectiveness of this type of learning is mainly affected by the load of visuospatial information on working memory in a dual-task context. Thus, this dual-task study potentially informs about the structure of the working memory component that coordinates dual-tasking.

## Task switching paradigms, theories, and executive functioning

Task switching refers to a multitasking situation where two or more tasks are presented sequentially without temporal overlap (e.g., Monsell, 2003; Kiesel et al., 2010). Contrasting with most of the studies on dual-tasking, the stimuli presented in task switching situations afford not only the currently relevant task but also the other task(s). For instance, participants may be presented with colored shapes as stimuli and frequently alternate between judging the color (Task A) or the shape (Task B). Another frequently used experimental protocol requires switching between purely semantic tasks, such as when participants judge the magnitude (Task A) vs. the parity (Task B) of stimulus digits. In single-task blocks, either Task A or Task B is presented exclusively. In mixed blocks, participants are confronted with both tasks either in a pre-specified task sequence such as AABBAABB (i.e., alternating runs paradigm; Rogers and Monsell, 1995) or with a random task sequence and a task cue that precedes or accompanies stimulus presentation (i.e., task cueing paradigm; Meiran, 1996). In these mixed blocks, the tasks can either repeat from one trial to the next (i.e., task repetitions) or switch (i.e., task switches), and two types of performance costs can be assessed. First, *mixing costs* are defined as the difference between the mean performance in trials with task repetitions in mixed blocks and the mean performance in single-task blocks (Koch et al., 2005; Rubin and Meiran, 2005). Second, *switch costs* are defined as the difference between the performance in task switch trials and the performance in task repetition trials within the mixed blocks (Rogers and Monsell, 1995).

The most prominent theoretical issue in task switching research has been the question of the origin of switch costs, particularly of so-called residual switch costs that are consistently found even after long preparation intervals during which participants have foreknowledge about the identity of the upcoming task. Although some accounts attribute residual switch costs to the duration of an executive process of task-set reconfiguration, occurring after encoding of the task stimulus (Rogers and Monsell, 1995; Rubinstein et al., 2001), there seems to be broad consensus that at least part of residual switch costs reflect priming from previous execution of the other task (e.g., Allport et al., 1994). In this regard, particular interest has been devoted to the role of task-set inhibition. Although the precise role of task-set inhibition concerning the residual switch costs is still unclear, convincing evidence for task-set inhibition is seen in the *N-2 task repetition effect* (a.k.a. the backward inhibition effect), found in task switching protocols that involve three different tasks (i.e., Tasks A, B, and C). The N-2 task repetition effect refers to the finding that the final trial of an ABA task sequence tends to be associated with slower responses than the final trial of a CBA sequence (Mayr and Keele, 2000), as would be expected if performance suffered from inhibition of the task-set for Task A in the former but not (or less so) in the latter case. Another major point in the task switching literature refers to attempts of specifying the processes involved in task preparation. Effective task preparation has been inferred from findings of improved performance when the preparation interval that precedes the presentation of the imperative stimulus is increased (Rogers and Monsell, 1995; Meiran, 1996). The precise processes involved in task preparation have proved difficult to determine, however (overviews in Karayanidis et al., 2010; Kiesel et al., 2010). More recent developments in task switching research refer to effects of task switching practice (e.g., Minear and Shah, 2008), individual differences (e.g., von Bastian and Druey, 2017), or the comparison of voluntary task selection with instructional task cuing (e.g., Arrington and Logan, 2004).

## Task switching studies in the present research topic

Articles included in the present research topic contribute to our understanding concerning the classical questions of task-set inhibition and task preparation as well as concerning practice, age-related differences, and voluntary task selection. As regards task-set inhibition, Schuch, using a diffusion model analysis, specified that older adults are not generally impaired in task inhibition in comparison to younger adults. Alternatively, there are age differences in dealing with task inhibition as reflected by differences in the speed-accuracy trade-off between these age groups. Jost et al. investigated whether task dominance determines backward inhibition. The results of their study showed that inhibition was stronger for more dominant tasks, suggesting that the amount of inhibition is adjusted in a context-sensitive manner. Concerning task preparation, Kleinsorge and Scheil presented redundant pre-cues that constrain the number of possible tasks from four to two before the task was cued unambiguously (replicating previous findings of an advantage of such pre-cuing; Kleinsorge and Scheil, 2015) and analyzed spontaneous eye blink rates. Changes in the eye blink rate during the initial part of the experimental session were correlated with pre-cuing benefit. Distinguishing between the preparation of perceptual and non-perceptual task processes, Wendt et al. focused on situations in which tasks differed regarding their perceptual demands of stimulus selection. Intermixing trials of a probe task they found evidence for preparatory adoption of task-specific attentional sets, that is, for focusing or defocusing of visual attention depending on the stimulus selection demands of a likely upcoming task. Wendt et al., by contrast, investigated preparation in the absence of a difference in perceptual demands between tasks. Analyzing task switching performance across six consecutive sessions, they extended previous evidence suggesting that task switching practice results in a speed-up of the preparation to non-perceptual preparatory processes. This study also introduced a probe task method—similar to the one applied by Wendt et al.—to research on task switching practice.

Buttelmann and Karbach's as well as Kray and Fehér's focus of interest is on age-related effects on task switching practice. Buttelmann and Karbach review the findings of training interventions and transfer in early and middle childhood, revealing substantial plasticity for different aspects of cognitive flexibility. Kray and Fehér assess the transferability of improved task switching performance after practice in young and older adults. Their findings suggest that the requirement to resolve interference between tasks is critical for the occurrence of transfer particularly in the elderly. A comparison of voluntary and instructed task selection—concerning the impact of task-specific action effects—was made by Sommer and Lukas. Finally, Moon et al. investigated interruptions of a visuo-tactile task by a second task that also involved visual and haptic stimuli, providing evidence for a helpful role of redundant haptic information in reducing the cost of interruption.

## Summary

In sum, the present research topic combines recent research in dual-tasks and task switching, focusing on the impact of executive functioning in these types of multitasking situations. In addition to the specific research issues addressed by the individual contributions, this collection of studies nicely shows the diversity of theoretical questions and methodological approaches in contemporary cognitive-neuroscientific research in this area.

## Author contributions

All authors listed have made a substantial, direct and intellectual contribution to the work, and approved it for publication.

### Conflict of interest statement

The authors declare that the research was conducted in the absence of any commercial or financial relationships that could be construed as a potential conflict of interest.

## Footnotes

^1^To avoid confounds with, for example, how many S-R translations need to be maintained in working memory, a third condition is sometimes employed where in each trial only one stimulus is presented, but potentially stimuli from both tasks can occur within one block (so called mixed blocks or heterogeneous single-task blocks; e.g., Schumacher et al., 2001; Strobach et al., 2014; Janczyk et al., 2015).
